# The effect of *Demodex*-associated *Bacillus oleronius* proteins and hyperosmolarity on a human conjunctival epithelial cell line

**DOI:** 10.1371/journal.pone.0350103

**Published:** 2026-05-22

**Authors:** Nikhil Sharma, Eilidh Martin, Edward Ian Pearce, Suzanne Hagan

**Affiliations:** 1 Department of Vision Sciences, Glasgow Caledonian University, Glasgow, Scotland, United Kingdom; Save Sight Institute, AUSTRALIA

## Abstract

**Purpose:**

Demodex mites act as a carrier vector for the bacterium *Bacillus oleronius*. Antigenic proteins from this bacterium are capable of producing an inflammatory response. This study investigated the potential effects of Demodex spp.-associated *Bacillus oleronius* proteins and dry eye-induced hyperosmolarity on an immortalised human conjunctival epithelial (Chang) cell line.

**Methods:**

The Wong Kilbourne derivative of the Chang (WKD) HeLa-modified conjunctiva-derived epithelial cell line was cultured in a series of NaCl-hyperosmolar conditions with various concentrations ranging from 350mOsm/L to 550mOsm/L. Chang cells were then exposed to semi-purified *B.oleronius* proteins at 2 µg and 6 µg either alone or in conjunction with dry eye-induced hyperosmolar (HO) stress at 2 µg + HO500 mOsm/L and 6 µg + HO500 mOsm/L. The cell viability was assessed with the RealTime-Glo MT cell viability assay.

**Results:**

Compared to controls, the cell viability appeared to decrease in a dose-dependent manner. B. *oleronius* concentration at 6 µg showed significantly lower cell viability than both controls (p < 0.001) and 2 µg (p < 0.001). When 500mOsm/L was added to the 2 µg and 6 µg concentrations, the lowest cell viability was observed (p < 0.001). At 24 hours, the *B.oleronius* 2 μg combined with HO500 resulted in 38% viable cells, compared to 81% viability in *B.oleronius* 2 μg alone (p < 0.001). Similarly, the *B.oleronius* 6 μg combined with HO500 showed only 23% metabolising cells compared to 60% viability cells in *B.oleronius* 6 μg alone (p < 0.001).

**Conclusion:**

The results suggest that the presence of bacterial proteins and hyperosmolarity have a dose and time-dependent effect on cellular viability. These experiments suggest that patients affected with the comorbidity of dry eye disease and *Demodex* blepharitis could experience more severe signs than those with *Demodex-*associated blepharitis alone.

## Introduction

The eyelids and conjunctiva are the habitat for a wide range of microbes which, under normal conditions, do not cause disease [1]. The prevalence of these microbes on the lids and ocular surface can be influenced by age, contact lens wear, inflammatory conditions (such as blepharitis) and systemic conditions, e.g., diabetes [2].

Two species of parasitic mites, *Demodex folliculorum* and *Demodex brevis,* are known to inhabit the eyelids and sebaceous glands, respectively [3]. *Demodex* mites play an aetiological role in contributing to various ocular surface diseases, including chronic blepharitis, dry eye disease (DED), meibomian gland dysfunction (MGD) and chalazion (meibomian cyst), amongst others. *Demodex* mites have been reported to activate inflammatory cascades, even after their death, due to the release of a bacterium (*Bacillus oleronius*), which is present inside the mite [4]. Kuhnigk *et al*. [5] isolated these bacteria from the hindgut of the termite Reticulitermes santonensis (Feytaud). Lacey *et al.* [6] then isolated *B. oleronius* from *Demodex folliculorum* and reported that two antigenic proteins (of sizes 62 kDa and 83 kDa) from the bacteria were capable of stimulating inflammatory responses. Further investigation by Li *et al.* [7] reported a correlation between serum immunoreactivity of *Bacillus* proteins and *Demodex* infestation.

*Demodex* mites have also been proposed to play an aetiological role and/or a risk factor in recurrent chalazion, although causality has not yet been established [8]. Chalazion is a chronic inflammation of the eyelid caused primarily by blockage of the meibomian gland, resulting in the formation of a red, painless lump [9]. Although the pathological mechanism remains unclear, studies have reported an association between *Demodex* and chalazion [10,11]. Moreover, it has been described that mechanical blockage of the meibomian glands by *Demodex* mites can stimulate a lipo-granulomatous reaction and follicular distension, leading to the development of chalazion [12]. Furthermore, *Demodex* could act as a vector in transferring bacteria, which could also lead to chalazion [12]. A similar hypothesis was proposed previously, where researchers suggested the role of an unknown antigen from diseased eyelids which caused conjunctival inflammation in ocular rosacea [13].

Tear hyperosmolarity is one of the core mechanisms in the vicious cycle of DED [14]. To understand the molecular mechanisms involved in DED, corneal and conjunctival cell lines have been extensively used in *in vitro* studies [15,16]. Since Demodex blepharitis infestation is considered as a co-morbidity of dry eye disease (DED), previous investigations have explored the effect of *Demodex*-associated *Bacillus* proteins on corneal epithelial cell lines [17,18]. The life cycle of *Demodex* is around two weeks, and it is postulated that upon the death of a *Demodex* mite, symbiotic bacteria from the mite may be released into the surrounding structures [4]. Since the conjunctiva is a crucial site for inflammatory reactions occurring on the ocular surface, an improved understanding of conjunctival epithelial cell function is necessary, due to its complex defence mechanisms [19]. However, to the best of the study team’s knowledge, no published study has explored their effect, either alone or in conjunction with DED-induced hyperosmolarity, on a conjunctival epithelial cell line. Therefore, this study aimed to investigate the potential effects of *Demodex*-associated *B. oleronius* proteins and dry eye-induced hyperosmolarity on a human immortalised conjunctival epithelial (Chang) cell line.

## Methods

### Cell culture

The immortalised human conjunctival epithelial cell line (Wong-Kilbourne derivative clone 1-5c-4, also termed “Chang cells”) was obtained from the American Type Culture Collection (ATCC, Virginia, USA), [20–22] and cells from passages P11 to P33 were used in all of the experiments. The Chang cells were cultured in Dulbecco’s Modified Eagle Medium (DMEM) + GlutaMax™ (Gibco, Paisley, Scotland, UK), supplemented with 10% FBS (Gibco) and 1% Pen/Strep (10,000 Units/mL penicillin and 10,000 µg/mL streptomycin, Gibco). Cells were cultured as a monolayer in T-75 filter-cap cell culture flasks (Sarstedt, Leicester, UK) in 20 ml of complete media at the standard conditions of 37°C, 5% carbon dioxide (CO_2_) and 95% humidity. The cells were passaged every 3–7 days and were observed under an inverted microscope for morphological changes (Nikon Eclipse TS-100, Tokyo, Japan).

### Hyperosmolarity experiments

For hyperosmolarity assays, Chang cells were seeded at 1 x 10^5^ cells per well in 12-well culture plates overnight (25). Cells were then exposed to hyperosmolar solutions of sodium chloride (5 mol L^-1^) NaCl; Sigma-Aldrich, Gillingham, UK) to determine how increasing NaCl concentrations affected Chang cell morphology and proliferative ability. A 2 ml sample of DMEM media was taken to determine the baseline osmolarity of the solution, using the Scoutpro (Tukera, formerly known as TearLab osmolarity system, TearLab, California, USA). An average of at least two media osmolarity readings were taken, prior to each experiment. Cells were then exposed to a range of hyperosmolar media to determine the most suitable conditions, ranging from 350 mM to 550 mM. All hyperosmolarity experiments to observe visual changes in cellular morphology were performed in duplicate and were repeated on three occasions.

### *Bacillus oleronius* preparation and protein extraction

A certified *Bacillus oleronius* strain [5] (strain designation – DSM 9356 [RT10], ATCC) was cultured in nutrient broth (Oxoid, Massachusetts, United States) at 30°C for 48 hours at 180 rpm. The *B. oleronius* culture and protein extraction methods have been adapted from previously published protocols [6,7,17,18]. Following the 48-hour incubation period, the bacteria were harvested by centrifugation at 4000g for 20 minutes (Sigma Sciquip 4K15, DJB Labcare Ltd, Buckinghamshire, UK). The supernatant was discarded, and the cells were washed twice with phosphate buffered saline (PBS). The cell pellet was then resuspended in 0.2% Triton X-100 (Merck Life Sciences UK Ltd, Gillingham, UK) and 2 ml of Lamberts Breaks Buffer [10 mmol L^-1^ KCL, 3 mmol L^-1^ NaCl, 4 mmol L^-1^ MgCl_2_, 10 mmol L^-1^ piperazine-N, N’ – bis (2-ethanesulphonic acid)] to solubilize the outer membrane proteins [7]. Protease inhibitor cocktail consisting of 10 µg mL^-1^ each of leupeptin, pepstatin A, aprotinin and N-α-p-tosyl-L-Lysine chloromethyl ketone hydrochloride (TLCK, Merck Life Sciences UK Ltd, Gillingham, UK)) were also added. The cell suspension was placed on ice and was sonicated for two 10 seconds blasts, using a soniprobe sonicator (Soniprep 150, Orpington, UK). The resultant sonicate was centrifuged at 6000g for 2 minutes at 4°C, to separate proteins from cellular debris. The sonicate was stored at −80°C until use.

### Cell viability assay

To observe the effects of hyperosmolarity on the Chang cells, the RealTime-Glo MT Cell Viability Assay (RTGLO, Promega, Madison, Wisconsin, United States) was used to measure the number of viable cells in real time. A trial experiment was performed to determine the assay linearity. This was done to determine the linear range of different cell densities over time and ensure that the results obtained were within the range of the assay. All of the hyperosmolar RTGLO experiments were performed as quadruplicates and were repeated three times in a white 96-well microplate (Greiner Bio One Ltd, Stonehouse, UK). The osmolarity of DMEM, as measured by the Tear-Lab test cards, was referred to as the “control” (or baseline), meaning healthy untreated cells in the usual control media. The “negative control” referred to wells containing complete media and the RTGLO reagents, but without the Chang cells present. This negative control also acted as a control to measure background luminescence.

### Cell toxicity assay

CellTox Green Cytotoxicity Assay (CTG, Promega) was used to assess hyperosmolarity-induced cell death of Chang cells. CTG uses a cyanine dye that binds to the DNA of cells with compromised cell membranes (or dead cells), but is excluded from live cells [23]. Increased fluorescence corresponded to the number of dead cells. All experiments were performed in quadruplicate wells, repeated twice in a black 96-well microplate (Greiner Bio-One Ltd). As before, the term “control” referred to Chang cells maintained in normal complete media (DMEM + 10% FCS). “Negative control” referred to wells containing complete media and the CTG reagent, but without the Chang cells. A positive/lysis control was included using Chang cells that were pre-treated with a lysis reagent which was added 30 minutes before the baseline reading (T0). For the remaining wells, 2 µl of CTG was added to 2 ml of each of the different hyperosmolar experimental conditions. The fluorescence was measured with the FLUOstar Omega microplate reader (BMG Labtech, Germany) after 1 minute of orbital shaking at 700–900 rpm, using an excitation wavelength of 485–500 nm and emission of 520–530 nm.

### Statistical analysis

The distribution of the data was assessed using the Shapiro-Wilk test. Data following a normal distribution was analysed using parametric tests. A one-way analysis of variance (ANOVA) was conducted to compare the group means. Following a significant result from the ANOVA, a Levene’s test was conducted to check the homogeneity of variance. An independent sample t-test or Welsch’s t-test was conducted. For repeated measurements, a repeated measures ANOVA was conducted to compare the differences in the group means. A paired sample t-test was conducted to compare repeated measurements. Data deviating from normal distribution was analysed by non-parametric tests. A Friedman’s ANOVA was used to compare the differences among the groups. A post hoc Mann-Whitney U test was conducted. For repeated measures, a Kruskal-Wallis test was conducted and a post hoc Wilcoxon’s signed rank test was conducted for pair- wise comparisons. A p-value of less than 0.05 was set for statistical significance. The mean ± standard deviation (sd), median and interquartile range (IQR) were used for descriptive statistics wherever appropriate. Data analysis was carried out using SPSS (version 28) and graphs were generated with GraphPad Prism (version 9.5.1).

## Results

### Morphological changes due to hyperosmolarity

**Fig 1:**
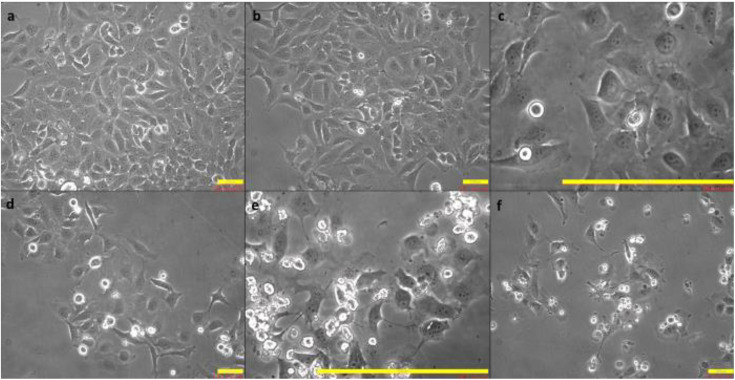
Figure showing cells in different hyperosmolar (HO) conditions observed at T24hrs, where a) control and b) HO350, both of the images show healthy and proliferating cells adhered to each other in the form of a colony. The cell membranes in both **c)** HO400 and **d)** HO450 appear to be disrupted, with occasional membrane blebbing and cytoplasmic swelling; e) and f) show the Chang cells in HO500 and HO550, respectively. Cells appeared stressed with membrane blebs, a greater number of apoptotic bodies and a relatively greater number of dead cells.

The morphology of the Chang cells appeared to vary in a dose- and time-dependent manner (Fig 1). The cells with higher concentrations of hyperosmolar media showed distinct morphological changes, such as stressed cells, disintegrated membranes, cell membrane blebbing, the formation of apoptotic bodies and a relative increase in the number of lysed/dead cells.

### Hyperosmolarity experiments: Dose-dependent changes in Chang cell viability

**Fig 2:**
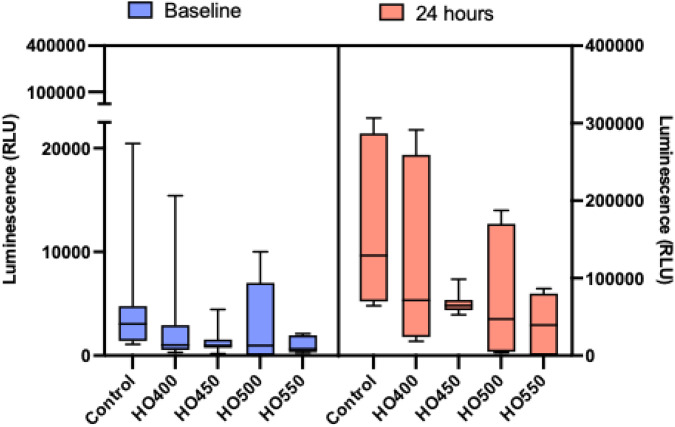
Boxplots comparing the cell viability at baseline vs 24 hours. At both time points (baseline and 24 hours), compared to controls, as the hyperosmolarity increased, the cell viability decreased (all p < 0.05). The right y-axis is on a different scale to that on the left, reflecting the increased cell numbers (growth) after 24 hours.

This experiment was performed to quantify Chang cell growth under different hyperosmolar concentrations. Compared to controls, the cell viability appeared to decrease in a dose-dependent manner, (Fig 2, all p < 0.01). As the hyperosmolarity increased, the apparent viability of the Chang cells decreased. It should be noted that in Fig 2, the right y-axis is on a different scale to that on the left reflecting the increased cell numbers after 24 hrs growth. Cell growth, however, was negatively influenced by the hyperosmolar media. That is, the higher the osmolarity, the lower the relative increase in cell numbers over time.

### Hyperosmolarity experiments: Dose-dependent changes in Chang cell toxicity

**Fig 3:**
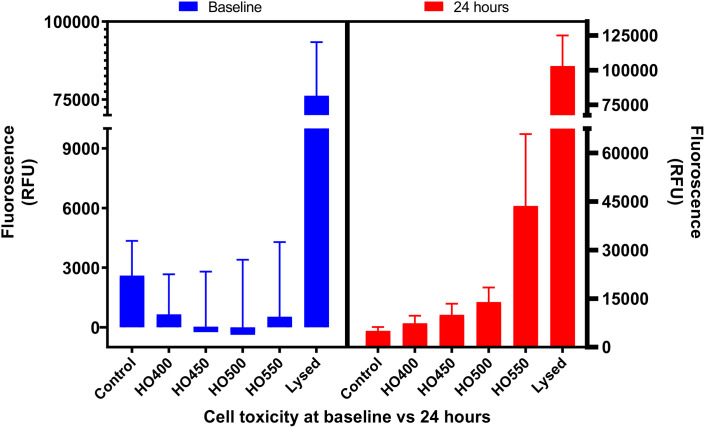
Graphs comparing the cell toxicity (mean ± SD) at baseline vs 24 hours. The cell toxicity increased in a dose-dependent manner over time. Compared to controls, there was a statistically significant increase in cell toxicity at 24 hours (all p < 0.01). Due to higher cell death in the positive control (lysed), the y-axis was split for a better visual comparison. Furthermore, due to intrinsic noise in the data arising from the CTG assay (background fluorescence), the RFU from negative controls (no cells, only media and assay) was first subtracted from all of the conditions. This explains the apparent negative values observed for HO450 and HO500 at baseline.

This experiment determined cell death as a function of hyperosmolarity, whereby higher fluorescence (RFU, Relative Fluorescence Unit) indicates higher cell toxicity. For a direct comparison of cell toxicity from baseline to 24 hours, Fig 3 shows the change in cell toxicity. It should be noted that in the figure, the right y-axis is on a different scale to that on the left. The cells in different hyperosmolar conditions continued to both multiply and die over the 24-hour period and therefore the total number of dead cells (measured by fluorescence) at 24 hours increased. This cell death was directly influenced by the hyperosmolar media, i.e., the higher osmolarity corresponded with increased cell death over time.

### Hyperosmolarity experiments: Time-dependent changes in cell viability

**Fig 4:**
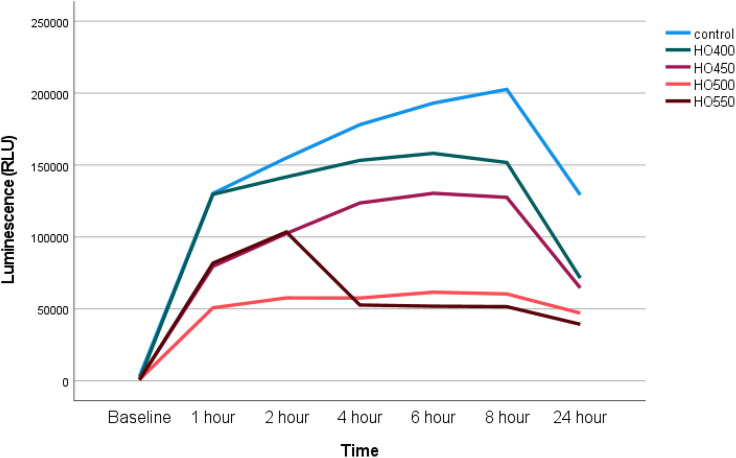
Line graph showing the cell viability (median) as a function of dose and time in different experimental conditions involving hyperosmolar media. At 24 hours, all conditions had fewer viable cells than the control (all p < 0.01).

The cell viability of different hyperosmolar experimental conditions (400mOsm – 550mOsm) was measured at a number of time points over 24 hours. A higher RLU indicates higher cellular metabolism and thus increased cell viability. An equal number of cells (n = 6000 cells/well) were seeded in all of the experimental conditions. However, the cell viability was affected in a dose- and time-dependent manner. In all of the experimental conditions, the highest cell multiplication was observed between T0 and T1hr. Except for HO550, a constant cell growth was observed until 8 hours (Fig 4).

### Hyperosmolarity experiments: Time-dependent changes in cell toxicity

**Fig 5:**
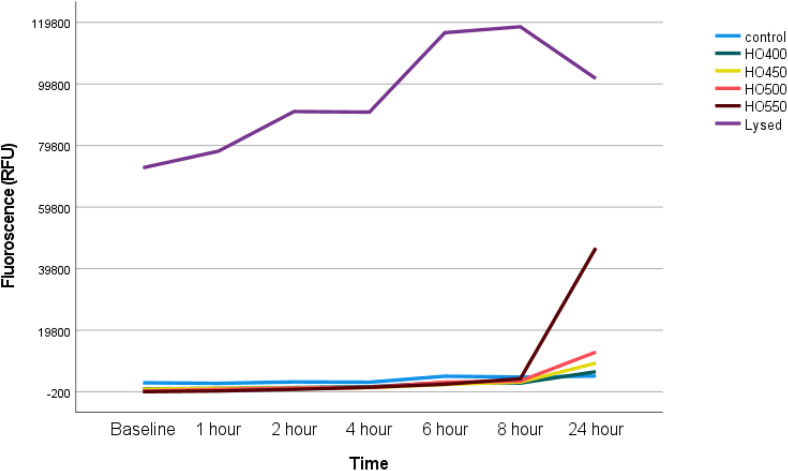
Line graph showing the cell toxicity (median) as a function of dose and time in different experimental conditions involving hyperosmolar media. Among the hyperosmolar conditions, HO550 showed the highest cell death (all p < 0.001).

This experiment assessed changes in cell *toxicity* under hyperosmolar stress as a function of time (Fig 5). Higher RFU values indicate higher cell death. Most of the cell death was observed after 8 hours in a dose-dependent manner.

### Dose-dependent effect of *Bacillus oleronius* proteins and hyperosmolarity on cell viability

**Fig 6:**
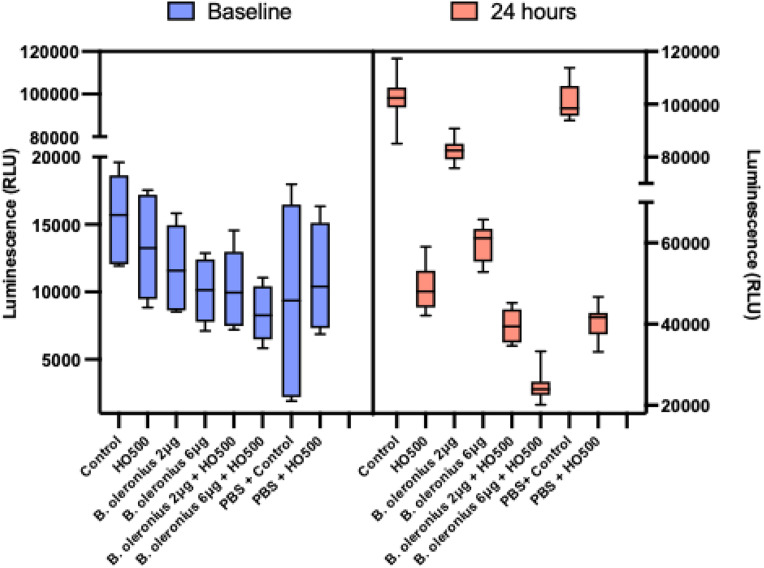
Boxplots comparing the changes in cell viability in different experimental conditions at baseline and at 24 hours. It should be noted that the y-axis on the right-hand side (24 hours) is on a different scale. This is because the cells in different media grew from baseline to 24 hours. This intentional scale difference is intended to illustrate a relative increase in cell growth from baseline to 24 hours.

The effect of *Bacillus oleronius* proteins on Chang cells was assessed, to determine the viability of Chang cells when exposed to 2 µg and 6 µg of the bacterial proteins alone, and in combination with hyperosmolar conditions, that is, 2 µg + HO500 (500mOsm/L) and 6 µg + HO500. The RLU for control and PBS + control (vehicle) did not show a statistically significant difference, either at baseline (*p > 0.05)* or at 24 hours (*p > 0.05),* indicating that the vehicle (PBS) used to dilute the bacterial proteins did not have any significant effect on Chang cell growth. The concentration of bacterial proteins, however, had a dose-dependent effect on cell viability, with 6 µg *B. oleronius* showing lower cell viability compared to control (p < 0.001) and to 2 µg *B. oleronius (p < 0.001,* Fig 6*).* Furthermore, adding HO500 in combination with the bacterial proteins had a greater effect on cell viability, with 6 µg + HO500 showing the lowest cell viability of all other conditions (all *p < 0.001*; Fig 6).

### Time-dependent effect of *Bacillus oleronius* proteins and hyperosmolarity on cell viability

**Fig 7:**
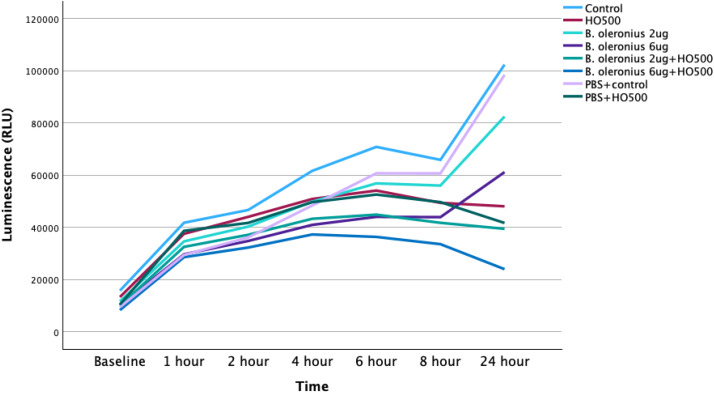
Line graph showing the cell viability (median) as a function of time and dose in different experimental conditions involving the bacterial proteins.

Chang cells were assessed for cellular metabolism as a function of time for each experimental condition (hyperosmolarity and *B. oleronius* proteins; Fig 7). The luminescence (RLU) was measured at different time points for 24 hours. A significantly higher number of viable cells were measured in controls (all p < 0.001). The cell viability between PBS+control and controls did not differ significantly, p > 0.05. *B. oleronius* 2 µg had significantly higher number of viable cells compared to *B. oleronius* 2 µg + HO500, p < 0.001. Similarly, *B. oleronius* 6 µg also had a significantly higher number of viable cells compared to *B. oleronius* 6 µg + HO500, p < 0.001 (Fig 7).

## Discussion

This study assessed the effect of Demodex-associated *B. oleronius* proteins, either alone or in conjunction with dry eye-induced hyperosmolar stress, on immortalised conjunctival epithelial (Chang) cells [21]. This study utilised a range of osmolar stresses (~350 mOsm/L – 550 mOsm/L) on Chang cells because of their greater resistance to hyperosmolar stress *in vitro* compared to *in vivo* [24]. When the salt concentration in the extracellular fluid becomes greater than the intracellular fluid, the cells experience hyperosmotic stress [25]. The imbalance in solute concentration leads to water flux, and a reduction in cell volume [14]. When the osmoadaptive responses fail to compensate for the solute imbalance, proapoptotic signalling is upregulated, leading to cell death [14]. This study modelled individual and combined effects of hyperosmolarity-induced DED and Demodex blepharitis-related proteins on conjunctival epithelial cell proliferation and differentiation.

In the current study, the morphological changes observed under the microscope over a 24-hour period showed dose-and time-dependent variations in cell shape, size, and a relative increase in the number of apparently dead cells. Due to no significant differences between cell viability in the 500mOsm/L and 550mOsm/L over 24 hours, only the former concentration was used in further experiments involving *B. oleronius* proteins. Moreover, 500 mOsm/L has also been identified as the lowest concentration capable of both stimulating expression of the chemokine (C-C motif) ligand 2 (CCL2)/monocyte chemoattractant protein-1 (MCP-1) [26]. MCP-1, with its C-C chemokine receptor 2 (CCR2), is an inflammatory chemotactic factor for recruiting monocytes [27]. Previously, MCP-1 levels have been reported to increase in the tears of DED patients [28], as well as in a dose-dependent manner in conjunctival cells exposed to hyperosmolar conditions *in vitro* [29].

The current study showed that the viability of Chang cells appeared to decrease in a dose- and time-dependent manner. Compared to controls, this effect was more profound at 24 hours, with a 45%, 50%, 64% and 70% reduction in metabolising cells exposed to HO400 mOsm/L, HO450 mOsm/L, HO500 mOsm/L and HO550 mOsm/L, respectively (all p < 0.01). Other hyperosmolarity studies on Chang cell viability have also shown a reduction in the number of metabolising cells. For example, a study by Clouzeau *et al.* [30] reported an almost 50% reduction in cell viability at HO500 mOsm/L. Another study reported only a 16% loss in cell viability at 500mOsm/L [31]. Although in the latter study, Warcoin et al. [26] reported that 550mOsm/L and 600mOsm/L reduced cell viability further, at 71% and 52%, respectively. The variations in cell viability in these studies could be attributed to the different assays employed [31]. Findings from *in vitro* studies have enhanced the understanding of the molecular mechanisms behind the pathogenesis of DED, by mimicking the short and long-term changes in DED. Previously, hyperosmolar stress studies on corneal and limbal cell lines have demonstrated the expression and production of pro-inflammatory markers, such as matrix metalloproteinases (MMPs 1, 3, 9 and 13), interleukin-1β (IL-1β), tumor necrosis factor α (TNF-α), and the C-X-C chemokine, IL-8, in a concentration-dependent manner [24]. Furthermore, studies conducted on corneal epithelial cells have shown that hyperosmolar stress-induced apoptosis occurs via a cytochrome c-mediated death pathway [32].

The effect of hyperosmolarity on cell death was demonstrated by using the CellTox green assay. Initially, the baseline cell toxicity for controls (untreated cells) was approximately 4%, while it was less than or equal to 1% in HO400, HO450, HO500 and HO550. However, a dose- and time-dependent effect was observed after 24 hours, in which the controls exhibited a 5% cell toxicity, 6% in HO400, 9% in HO450, 13% in HO500 and 46% in HO550. This exponential increase in cell death in a time- and concentration-dependent manner indicated the toxic effect hyperosmolar stress has on Chang cells over a period of 24 hours. One of the possible explanations for a lower cell toxicity in HO-exposed Chang cells compared to controls at baseline could be attributed to the greater noise in the data arising from background fluorescence.

The cell viability of Chang cells was also investigated by adding *B. oleronius* proteins either alone, or in combination with HO500 (500mOsm/L). In order to evaluate their effects on cell viability, cells were exposed to 2 µg/mL and 6 µg/mL of semi-purified bacillus proteins, respectively. In addition, to mimic dry eye conditions, 500 mOsm/L of hyperosmolar media was combined with 2 µg and 6 µg of semi-purified bacillus proteins. At 24 hours, compared to controls, there was a dose-dependent reduction in cell viability, with HO500 showing a 52% decrease, B. *oleronius* 2 µg (alone) showed a 19% decrease, B. *oleronius* 6 µg (alone) showed a 42% decrease, B. *oleronius* 2 µg + HO500 showed a 62% decrease and B. *oleronius* 6 µg + HO500 showed a 76% decrease (all p < 0.001). A previous study on a human corneal epithelial cell line (hTCEpi) also demonstrated a dose-dependent reduction in cell growth, whereby 2 μg/mL of *Bacillus* proteins inhibited the growth rate by 26% (p = 0.018) and, at 6 μg/mL, the bacterial proteins inhibited cell growth by 75% (p = 0.0003) [18]. Furthermore, compared to controls, exposing hTCEpi cells to *Bacillus* proteins has shown a dose-dependent increase in cell mobility (3.6-fold with 2 µg/mL, p < 0.001 versus 15-fold with 6 µg/mL, p < 0.0001), invasiveness (1.7-fold; p = 0.003) with 2 μg/mL of the *Bacillus* protein and by 1.8 fold (p = 0.01) with 6 μg/mL) and wound healing [18].

The *in vitro* analysis in this study demonstrates that dry eye-type conditions (i.e., hyperosmolarity) and the presence of *Demodex*-associated bacterial proteins leads to a dose-dependent reduction in cell viability. Therefore, it could be hypothesised that, when combined, these factors may have an additive effect. Hence, it could be extrapolated from these findings, that patients suffering from both DED and *Demodex* blepharitis may experience more severe signs and symptoms than for each condition separately. This hypothesis is in conjunction with the recently published clinical studies, which reported that ocular signs and symptoms were worse in patients with *Demodex* and DED [33,34].

### Limitations

It has been reported that the Chang cell line has been contaminated by the HeLa cells, raising concerns regarding its tissue origin and physiological relevance. However, the Chang cell line model retains epithelial-like characteristics and remains widely used in ocular surface research. Findings derived from this cell line should be interpreted with caution, particularly when extrapolating to native conjunctival biology as Chang cells may not fully recapitulate the functional phenotype of primary conjunctival epithelium [35]. Primary conjunctival epithelial cells represent the most physiologically relevant in vitro model, whereas Chang cells offer experimental reproducibility but may exhibit altered biological behaviour, particularly in light of their reported HeLa contamination. Another shortcoming of the current study is the reliance on crude or semi-purified *B. oleronius* proteins. Due to this, the fluorescent-based assay could not be used to assess the toxic effect of Demodex-associated *B. oleronius* proteins. In this study, the *B. oleronius* proteins used were a crude extract and may have contained bacterial DNA. Therefore, the fluorescent signal would not only represent of the DNA of the dead Chang cells, but possibly also that of the bacterial DNA. Hence, the cell toxicity may not be a true representative of the actual toxic effect of *Bacillus* proteins on Chang cells.

Future research could explore the cytotoxic effect of these proteins on human conjunctival and corneal epithelial cells, their effect on the production of reactive oxygen species (ROS) and the use of scratch wound assays to ascertain the mobility of *B. oleronius-*treated conjunctival epithelial cells. Furthermore, the toxicity of *Demodex*-associated proteins (of more purified and of known molecular weight) on the ocular surface needs to be assessed in future studies.

## Conclusion and future work

In conclusion, these experiments show that *Demodex*-associated *Bacillus oleronius* proteins and hyperosmolar stress (as a basic *in vitro* model of DED), do have an additive effect in terms of reduced cell viability and potentially more cell death. This could mean that patients with both *Demodex* blepharitis and DED may have potentially more damaging effects on the ocular surface. Due to the differences in the physiological relevance of primary cells versus an immortalised cell line, the results provide insights within a simplified epithelial model rather than a direct representation of native conjunctival physiology and hence should be interpreted accordingly.
